# Support preferences among women with and without postpartum depression and anxiety disorder

**DOI:** 10.1186/s12889-025-24274-y

**Published:** 2025-09-12

**Authors:** Vanessa Zieß, Lara Seefeld, Amera Mojahed, Julia Martini, Eva Asselmann, Julia Schellong, Susan Garthus-Niegel

**Affiliations:** 1 Institute for Systems Medicine (ISM), Faculty of Medicine, Medical School Hamburg, Hamburg, Germany; 2 Institute and Policlinic of Occupational and Social Medicine, Faculty of Medicine, TUD Dresden University of Technology, Dresden, Germany; 3 Department of Psychotherapy and Psychosomatic Medicine, Faculty of Medicine, TUD Dresden University of Technology, Dresden, Germany; 4 Department of Psychology & Neuroscience, Dalhousie University, Halifax, Canada; 5 Department of Psychiatry and Psychotherapy, Carl Gustav Carus University Hospital, TUD Dresden University of Technology, Dresden, Germany; 6 Department of Psychology, Institute for Mental Health and Behavioral Medicine, HMU Health and Medical University, Potsdam, Germany; 7 Department of Childhood and Families, Norwegian Institute of Public Health, Oslo, Norway

**Keywords:** INVITE study, Postpartum depression, Postpartum anxiety, Counseling and treatment services, Service delivery mode, Preferences, Help-seeking

## Abstract

**Background:**

Some women struggle with mental health problems such as postpartum depression (PPD) or postpartum anxiety disorder (PAD) after giving birth. This can have a negative impact on the new mother, the infant, and the whole family. However, many women experiencing PPD and/or PAD go unrecognized and untreated. Since appropriate support is essential, efforts should be made to facilitate women’s help-seeking behavior. The purpose of this study was to improve the mental health of postpartum women by understanding their specific support preferences. To this end, the preferences for counseling and treatment services, as well as the service delivery mode among women with PPD, PAD, comorbid PPD and PAD, and women with neither PPD nor PAD were examined.

**Methods:**

In the cross-sectional study INVITE, mothers (*n* = 2,031) were interviewed via telephone about three to four months after birth. PPD was assessed using the Edinburgh Postnatal Depression Scale (EPDS), PAD was assessed using the anxiety scale of the Symptom-Checklist-90-Revised (SCL-90-R), and preferences for services and delivery modes were assessed using self-generated questionnaires. Analyses of covariance were performed to examine differences between the symptom groups.

**Results:**

All women preferred the support of *(family) midwives* and *family*,* friends*,* or colleagues* and to talk to someone *in person*. Analysis of covariance showed that, overall, women with PPD preferred all services less than women with neither PPD nor PAD. Furthermore, women with PPD preferred psychotherapeutic services (e.g., *inpatient clinic* and *outpatient clinic/treatment*) less, and women with comorbid PPD and PAD preferred professional and personal confidants (e.g., *midwife* and *women in the same situation*) less than all other women. Women did not differ in their preferences for service delivery mode.

**Conclusions:**

This study provides unique insight into postpartum women’s preferences for various services and delivery modes. Results showed that women differ in their preferences for services depending on their symptoms. This should be considered when making referrals, and postpartum support should be better tailored to mothers’ wishes and needs to improve help-seeking behavior and ultimately postpartum mental health.

**Supplementary Information:**

The online version contains supplementary material available at 10.1186/s12889-025-24274-y.

## Background

The time after giving birth is often a very stressful period for new mothers, including substantial hormonal changes and everyday challenges with the newborn. Mental health problems can occur, with nearly 18% of mothers worldwide experiencing symptoms of postpartum depression (PPD), which may last up to 12 months postpartum [1]. Another mental health disorder that can manifest after birth is postpartum anxiety disorder (PAD), which is less frequently explored (i.e., 2.2% of research on postpartum mental health) than PPD (i.e., 69.6%) [2–4]. However, with a prevalence of approximately 15 to 20%, it is at least as important to investigate as PPD [5, 6]. In some women, PPD and PAD are comorbid (i.e., prevalence 8.2%) [7] and symptomatology is often more severe and persistent in these women [8, 9]. Additionally, women with PPD may be at increased risk for an anxiety disorder in the future and vice versa [10].

If the mother’s symptoms of PPD and PAD are not treated, her risk for mental health problems in the future increases [11, 12]. In the case of her child, cognitive developmental delays [13, 14], impaired social-emotional development [15, 16], development of psychopathology, and long-term behavioral problems [17] are possible, among other things. Hence, support of women affected by postpartum mental health problems is important to prevent negative consequences for the whole family. Nevertheless, many affected women do not seek help [18–20]. One reason for this may be that women do not realize that they are experiencing symptoms of PPD or PAD [21–24]. Often, postpartum mental health problems are not even recognized by healthcare professionals [25]; and less than 10% of affected women are referred to specialist care [26], although empirical evidence for effective treatment is growing [3, 27–30]. In addition, women are not adequately informed about the support services available to them or do not know which service is appropriate for their symptoms [31–33]. This underutilization may also indicate that support services are not sufficiently responsive to women’s needs and preferences. For these reasons, it is important to determine how to facilitate the path to appropriate counseling and treatment services after birth, thereby closing the gap between the need for help and actual help-seeking.

Therefore, it is of utmost importance to research the service preferences of those affected, to derive targeted interventions. Most studies reporting the kind of support desired by women with PPD or PAD used qualitative methodologies [34–37]. In particular, support from the social environment, such as partner, family, and friends, was frequently mentioned [34–37]. Contact with peers, i.e., other women with children or new mothers experiencing the same mental health problems, was also seen as an important source of support for affected women [34–37]. Another service that women valued was support from (mental) health professionals [36, 37]. However, few studies have examined preferences for a range of different service options, and the results of these studies are sometimes contradictory. In one study, support in a physical healthcare setting was preferred to a mental healthcare setting [38], while the opposite was true in another study [39]. In addition, in most studies, mental health providers were highly popular among affected women [38–40], with one exception where informal help from family, friends, and colleagues was preferred above all [41].

As women with PPD, PAD, and comorbid PPD and PAD may have distinct preferences for services and delivery modes, it is crucial to analyze each group separately and compare them in order to provide targeted support that may increase the likelihood of help-seeking.

Although some women may be below the clinical cut-off level, they may still experience debilitating depressive or anxiety symptoms. For these women, the provision of appropriate support services is also relevant as they too may need effective and suitable treatment, especially for the prevention of more serious symptomatology. Therefore, the extent to which women with psychopathological symptoms and women experiencing no or subliminal symptoms differ in their preferences is crucial to investigate. The women below the clinical cut-off can therefore be considered as a baseline group for better interpretation of the results. Little research has been done on this to date. In previous literature, differences in preferences between women with and without postpartum mental health disorders could be found [40–42]. This suggests that different types of support may be needed. For example, women with PPD were found to have more negative attitudes towards professional help (i.e., psychological counseling or psychotherapy) than women without or with subliminal symptoms [42]. At the same time, women with PPD and/or PAD were less likely to turn to informal sources of help (e.g., partner or friend) than women below the cur-off score [41]. In contrast, in a study of Israeli women, there were no differences in preferences between women with and without or with subliminal PPD, except for community treatment centers, which were preferred by women without or with subliminal symptoms [40]. However, research on this topic is inconsistent and results of different studies are contradictory. Further research is needed, as no studies were found regarding differences in preferences between women with PAD alone, comorbid PPD and PAD, or women without these symptoms.

Moreover, research on and development of e-mental health interventions continues to increase and is intended to facilitate and supplement treatment processes in the future. However, it is questionable to what extent this is desired by patients. Previous literature suggests that women with PPD prefer personal face-to-face contact with professionals above all [33, 36]. Telephone support was less preferred by women with PPD [38], but positively evaluated when it was timely and accessible [36]. Overall, opinions on offers on the internet are mixed. There is evidence that women with PPD are very interested in e-mental health, such as communicating with a mental health professional via chat or video chat (40%), participating in blog exchanges (60%), chatting with other mothers (44%), or participating in an expert-moderated chat room (65%) [43]. In contrast, e-mental health interventions were rated rather poorly by affected women in some other studies [33, 38–40] and only one of those studies examined differences between women with and without psychopathological symptoms [40]. In this study, no significant differences in preferences could be found for any delivery mode (i.e., in person, video conference, telephone, chat with professional or peer, phone application, website with guidance from professional) [40].

The purpose of the present study was to explore women’s preferences for postpartum counseling and treatment services and service delivery mode. Understanding the types of support services that women with different symptomatology want to use can provide specific recommendations and individualized help that is tailored to their wants and needs. This study extends previous research by examining a wide range of services offered by different providers, in different settings, and of varying delivery mode. As previous studies have focused nearly exclusively on women with PPD, the current study aims to involve women with PAD and comorbid PPD and PAD as well. It was investigated if and how women with PPD, PAD, comorbid PPD and PAD, and women with subliminal or no psychopathological symptoms differ from each other (a) in their overall ratings of all counseling and treatment services, (b) in their preferences for certain groups of services, (c) in their overall ratings of all service delivery modes, and (d) in their preferences for certain groups of service delivery modes.

## Methods

### Design

The data used in this study derive from the cross-sectional study INVITE (INtimate partner VIolence Treatment PrEferences) [44]. INVITE examines the preferences for, and barriers to, counseling and treatment services for women in the postpartum period. The aim is to investigate women’s health and factors that facilitate access to appropriate services, especially in the case of mental health problems. For this purpose, a standardized telephone interview lasting for approximately one hour was conducted with the participants at three to four months postpartum.

Recruitment for the study took place from November 2020 to the end of October 2023 at various maternity hospitals and freestanding birth centers with different levels of specialization for high and low risk pregnancies in the Dresden area – more specifically at birth information events, midwife (antenatal) appointments, and the maternity ward. The participants were personally recruited through student assistants who approached them with information about the study. This ensured that every eligible woman in Dresden was spoken to. During lockdown periods caused by the Covid-19 pandemic, student assistants were unable to recruit in person. As a result, study materials could only be distributed by hospital staff, which likely affected recruitment numbers. This is because hospital staff had less time to talk to the women and less background knowledge about the study than the student assistants. After giving written consent, women received 5€ immediately and another 15€ after the conduction of the telephone interview as monetary compensation. Detailed information on the study design and recruitment process can be found in the study protocol [44].

Data collection and management was facilitated using Research Electronic Data Capture (REDCap), a secure, web-based application for data capture as part of research studies, hosted at the ‘Koordinierungszentrum für Klinische Studien’ at the Faculty of Medicine of the Technische Universität Dresden [45, 46]. This study involved human participants. It was approved by the Ethics Committee of the Technische Universität Dresden. All participants provided written informed consent to participate in this study. The current study is based on version 2 of the quality-assured data files of the INVITE study (data extraction on 03/06/2022).

### Participants

**Fig. 1:**
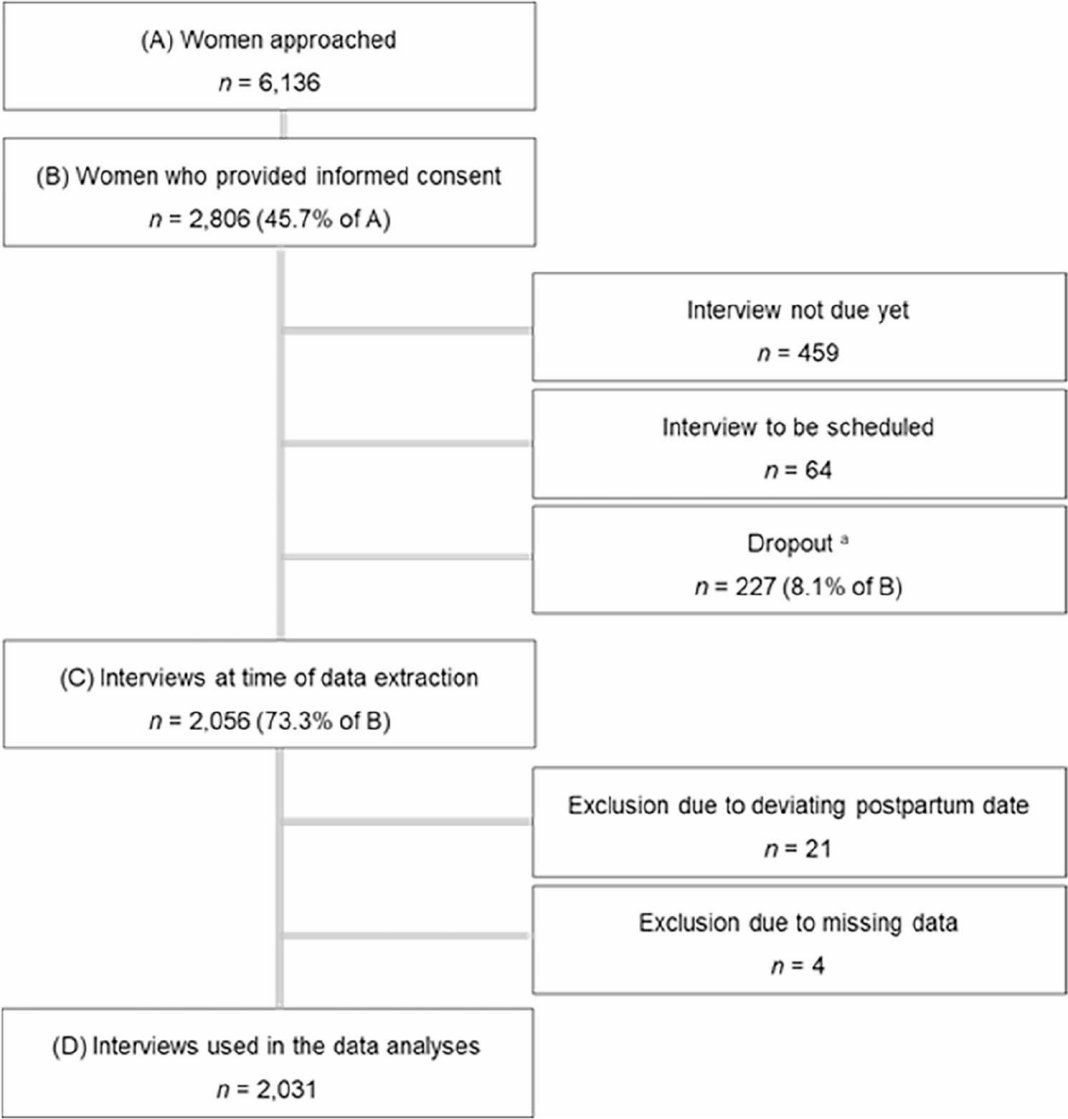
Study population and retention rate Note. The flowchart includes the response rate, dropouts, exclusions, and final sample size based on the recruitment between November 2020 and June 3rd, 2022.^a^ Due to withdrawn consent, a child older than six months, or because they could not be reached

The objective was to target a community sample of women giving birth in Dresden, Germany. Of the approximately 8,000 births that took place in Dresden during this period, we were able to approach 6,136 women [47]. Women who were younger than 16 years of age or who did not have sufficient language skills in German or English to take part in the study were excluded. At the time of data extraction, 2,806 women (45.7%) had given consent to participate in the study, and of them, 2,056 women (73.3%) had completed the interview. To obtain comparable results, women who completed the interview earlier than eight weeks or later than six months after birth were excluded (*n* = 21). Additionally, some women had to be excluded due to missing data in all variables of interest (*n* = 4). The retention of the study population is presented in Fig. 1.

### Measures

#### Assessment of postpartum depression

The Edinburgh Postnatal Depression Scale (EPDS), one of the most commonly used screening tools for postpartum depression symptoms, was used to assess PPD. The emotional state and well-being during the past week with symptoms specific to the postpartum period are measured. The scale consists of 10 items and the participants have to answer on a four-point Likert scale, with scores ranging between 0 and 30 [48–50]. To specifically research the preferences of women with mild symptoms, a cut-off score of 10, indicating minor depression, was chosen rather than a cut-off score of 13, indicating moderate to severe depression [38, 50]. The lower cut-off was considered more appropriate as it includes all burdened women and therefore has the potential to allow for early intervention. At this cut-off value, the EPDS has been found to have a sensitivity of 0.96 and a specificity of 1.0 for both minor and major depression [48]. The internal consistency of the EPDS was good in this sample (Cronbach’s α = 0.80).

#### Assessment of postpartum anxiety disorder

To assess PAD, the anxiety subscale of the Symptom Checklist-90-Revised (SCL-90-R) was used [51, 52], a validated and widely used diagnostic instrument, which measures psychological and physical symptoms associated with anxiety occurring over the past seven days. The SCL-90-R anxiety scale consists of 10 items with response options ranging on a five-point Likert scale with a minimum total score of 0 and a maximum total score of 40 [52]. For each participant, a mean score was calculated, which was then converted into normative T-values. A T-value greater than 60 is often chosen to identify individuals who are experiencing clinically relevant levels of symptoms that warrant further assessment or intervention and was therefore used as the threshold in this study [52]. The SCL-90 anxiety subscale can significantly differentiate between women with postpartum anxiety symptoms and non-affected women [53]. Thus, it is suitable to identify women who meet the criteria for PAD. In this sample, the internal consistency of the SCL-90-R anxiety scale was good (Cronbach’s α = 0.81).

#### Assessment of counseling and treatment service preferences

A self-generated questionnaire based on Simhi et al. [40] was used to assess preferences for counseling and treatment services of varying specialization [44]. For this purpose, affected women were asked to indicate on a four-point Likert scale whether they would choose the listed services or not. Non-affected women were asked to imagine that they are experiencing symptoms of PPD or PAD and to respond to the questions as if they were affected. However, the same set of instructions detailing a variety of sample symptoms (such as depressive mood or uncontrollable worries) was presented to all women. The questionnaire consists of 20 items, with scores ranging between 0 and 80. A framework model was utilized to categorize services that could potentially be used [44]. Services were categorized as social (e.g., self-help groups), psychosocial (e.g., consultation hotline), and medical services (e.g., family midwife). In addition, non-specific (e.g., offers for families in general) and specific services (e.g., trauma-specific psychotherapeutic outpatient treatment) were distinguished. Participants were also asked whether they would prefer a therapy of the mother alone or mother-child therapy when utilizing psychiatric or psychosomatic treatment. The various counseling and treatment services surveyed in the current study were selected specifically for the German support system and attempt to cover all available services provided in Dresden that are eligible for women with postpartum mental health problems [44].

To extract the most important independent factors of counseling and treatment service preferences, an exploratory Principal Axes Factor Analysis (PFA) with Varimax rotation was conducted. Examination of Kaiser’s criteria and the scree-plot yielded justification for retaining four factors with eigenvalues exceeding 1, which accounted for 49.97% of the total variance. One item, *religious institutions*, was excluded due to a factor loading below 0.30. The following main factors were derived: [1] Professional and Personal Confidants (e.g., *midwife* or *woman in the same situation*) [2], Communal and Psychosocial Services (e.g., *household help* or *psychosocial crisis service*) [3], Medical Services (e.g., *general practitioner* or *pediatrician*) [4], Psychotherapeutic Services (e.g., *day clinic for psychiatry or psychosomatic medicine* or *specialized trauma outpatient clinic*). PFA revealed a pattern that deviated from the theoretical framework model [44]. More detailed information is provided in an additional file (see Additional file 1). The internal consistency of the counseling and treatment service preferences questionnaire was good (α = 0.81). Using McDonald’s ω, the internal consistency of subscales ranged from unacceptable for professional and personal confidants (ω = 0.420), questionable for medical services (ω = 0.675), acceptable for psychosocial services (ω = 0.777), and good for psychotherapeutic services (ω = 0.815).

#### Assessment of service delivery mode preferences

To assess the preferred service delivery mode, a self-generated questionnaire was used [44]. The questionnaire consists of seven items and participants can answer on a four-point Likert scale, with a minimum total score of 0 and a maximum total score of 28, whether the different options meet their needs. Direct personal (e.g., *in person*), indirect personal (e.g., *via e-mail*), and impersonal (e.g., *through an app or online platform*) delivery modes were covered. Furthermore, a distinction was made between real-life and web-based options. In accordance with the assessment of counseling and treatment service preferences, participants were asked to indicate their preferences based on their current situation or imagine that they are experiencing the described psychopathological symptoms.

Again, PFA with Varimax rotation was performed to identify the main factors of service delivery mode preferences. Due to insufficient discriminatory power, the item *in person* was excluded from the mode of service provision preferences questionnaire. The analysis produced two factors explaining a total of 65.31% of the variance: [1] Direct Communication (*video conference* and *telephone call*) [2], Delayed Communication (e.g., *chat*, or *app or online platform*). More detailed information is provided in an additional file (see Additional file 2). The consistency of the remaining six items in the service delivery mode preferences questionnaire was acceptable (α = 0.74). Internal consistency for delayed communication was acceptable (ω = 0.776). Internal consistency for direct communication could not be calculated using McDonald’s Omega as less than three items were included in this scale. Therefore, interitem correlation was used, and direct communication was found to be within the recommended range of *r* =.15 to *r* =.50 (*r* =.442) [54].

### Data analysis

For the following data analyses, IBM SPSS Statistics (Version 28.0.0.0) was used. First, the data were checked for outliers. Univariate outliers were identified using boxplots displaying the interquartile range (IQR). Values three times greater than the IQR were considered extreme univariate outliers and five cases were identified. Using Mahalanobis distance, 23 cases were detected as multivariate outliers. Sensitivity analyses were then performed to control for the influence of all identified outliers. Subsequently, the main sociodemographic characteristics, potential confounding variables (maternal age, duration of residence in Germany, income, and parity), predictors, and outcome variables were examined descriptively. Furthermore, assumptions for all analyses were tested. Missing values of less than 20% in one scale were replaced by the woman’s mean value.

Four different groups were distinguished for the analyses: Women with PPD only, women with PAD only, women with comorbid PPD and PAD, and women with neither PPD nor PAD. For the classification into the different groups, the cut-off scores of the EPDS and SCL-90-R anxiety scale were used [48, 50–52].

Past studies have shown that maternal age, duration of residence in Germany, income, and parity are associated with postpartum help-seeking behavior and were therefore considered as potential confounders in this study [19, 55–58]. Those variables that correlated significantly with an outcome variable (counseling and treatment service or service delivery mode preferences) in Spearman correlation analysis were included in the respective analyses. To investigate the research questions, two one-way analyses of covariance (ANCOVA) were performed to investigate the differences between the total scores of the counseling and treatment service preferences as well as the service delivery mode preferences between all groups. To reveal the differences between all groups and their relation to the counseling and treatment service preferences sub-scores and the service delivery mode preferences sub-scores, two one-way multivariate analyses of covariance (MANCOVA) were applied. Assumptions for ANCOVAs and MANCOVAs were tested. As some assumptions for ANCOVA and MANCOVA were not confirmed (normal distribution of residuals, multivariate normal distribution, and linearity between predictor and outcome variables), all analyses were performed with bootstrapping using the bias-corrected and accelerated (BCa) method with 95% confidence intervals (CI). After the one-way ANCOVAs and MANCOVAs were conducted, post-hoc tests using Bonferroni-corrected mean comparisons based on the estimated marginal means were calculated. A significance level of *p* <.05 with a 95% confidence interval was used for all analyses. Due to missing values in individual variables, *n* varied slightly between analyses.

## Results

### Descriptive statistics

The final sample comprised *n* = 2,031 mothers with a mean age of 32.9 years (*SD* = 4.5). At the time of the interview, newborns were on average 12.5 (*SD* = 2.1) weeks old. In this study, 10.5% of the women had scores representing only PPD, 1.4% experienced only PAD, 4.4% experienced both PPD and PAD, and 83.6% experienced neither PPD nor PAD. Detailed sociodemographic characteristics of the sample are presented in Table 1.

**Table 1:** Sociodemographic characteristics of the sample

Characteristic	M (SD)	Range
Maternal age ^a^	32.9 (4.5)	16.8–51.3
Age of child ^b^	12.5 (2.1)	8.4–25.1
	*n*	%
Duration of residence in Germany ^c^		
< 5 years	47	2.3
5–10 years	52	2.6
> 10 years	64	3.1
Born in Germany	1,868	92
Partnership status		
Partner	1,989	97.9
No partner	42	2.1
Education		
≤ 10 years	528	26
> 10 years	1,502	74
Net income ^d^		
< 1,250 €	48	2.4
1,250 € – 2,249 €	240	11.9
2,250 € – 2,999 €	271	13.5
3,000 € – 3,999 €	525	26.0
4,000 € – 4,999 €	500	24.7
> 5,000 €	435	21.5
Parity		
Primiparous	1,057	52.1
Multiparous ^e^	974	47.9
1	753	37.1
2	181	8.9
3	27	1.3
≥ 4	13	0.6
Symptom groups		
Without PPD and PAD	1,698	83.6
PPD	214	10.6
PAD	29	1.4
Comorbid PPD and PAD	90	4.4

Note. *N* = 2,031

^a^ in years

^b^ in weeks

^c^ time since migration to Germany

^d^ per month and household

^e^ additional children to the newborn

Table 2 shows the descriptive statistics of support preferences for all items across all symptom groups. Among the various counseling and treatment services, women overall preferred (*family*) *midwife* and *family member*,* friend*,* or colleague* the most and preferred *religious institutions* the least. *In person* was the most popular service delivery mode, followed by *video conference* and *telephone call.* Women rated *app or online platform without guidance from an expert* as the least popular.

**Table 2:** Descriptive statistics of support preferences for individual items

	Service preferences ^a^	M (SD)	Mode preferences ^b^	M (SD)
Definitely	Midwife	3.7 (0.6)	In person	3.7 (0.5)
(4)	Family midwife ^c^	3.5 (0.7)		
	Family member, friend, or colleague	3.5 (0.8)		
	Women in the same situation	3.3 (0.7)		
	Gynecologist	3.3 (0.8)		
Rather yes	Outpatient clinic ^d^/treatment ^e^	3.2 (0.8)		
(3)	Life and family counseling ^f^	3.1 (0.7)		
	General practitioner	3.1 (0.9)		
	Trauma outpatient clinic	3.0 (0.8)		
	Pediatrician	2.9 (1.0)		
	Household help	2.9 (0.9)		
	Supervised parent group	2.8 (0.8)		
	Psychosocial crisis service	2.7 (0.8)		
	Self-help group	2.7 (0.9)		
	Day clinic ^d^	2.6 (0.9)	Video conference	2.6 (0.8)
	Social pedagogical family assistance	2.6 (0.9)	Telephone call	2.5 (0.7)
Rather no	Inpatient clinic ^d^	2.4 (0.9)		
(2)	Telephone counseling	2.3 (0.9)		
	Parent-child living or family accommodation	2.3 (1.0)	App or online platform with guidance ^g^	2.0 (0.8)
	Religious institutions	1.8 (0.9)	E-mail	1.8 (0.7)
Not at all			Chat	1.7 (0.7)
(1)			App or online platform without guidance ^g^	1.5 (0.6)

^a^ Counseling and treatment service preferences

^b^ Service delivery mode preferences

^c^ Offers additional support with everyday tasks and gives advice on childcare

^d^ In the department for psychiatry or psychosomatic medicine

^e^ By a registered medical or psychological psychotherapist

In addition to psychotherapeutic services, women were asked what type of therapy they favored. The majority of women preferred mother-child therapy (63.3%) over therapy of the mother (37.5%). Table 3 provides a detailed description of each symptom group.

**Table 3:** Descriptive statistics of preference for type of psychotherapeutic treatment

Type of treatment	Without PPD or PAD	PPD	PAD	Comorbid PPD and PAD
	*n* (%)			
Therapy of the mother	605 (35.6)	97 (45.3)	18 (61.2)	42 (46.7)
Mother-child therapy	1,092 (64.3)	127 (59.3)	13 (44.8)	53 (58.9)
Don’t know	186 (11.0)	14 (6.5)	1 (3.4)	6 (6.7)

Note. Both treatment options could be chosen

### Correlation analysis

Spearman’s rank correlation was computed to assess the relationship between covariates and outcome variables. There was a significant correlation between maternal age and the total score of counseling and treatment service preference (*r* =.048, *p* =.031). Therefore, maternal age was included as a confounder in the analyses (i.e., ANCOVA for the outcome variable total score and MANCOVA for the outcome variables sub-scores of counseling and treatment service preferences). For the total score of the service delivery mode preferences, a significant correlation with maternal age (*r* =.059, *p* =.008) and parity (*r* = −.062, *p* =.006) could be found. Again, maternal age and parity were used as confounders in the analyses (i.e., ANCOVA for the outcome variable total score and MANCOVA for the outcome variables sub-scores of service delivery mode preferences). More detailed information is provided in an additional file (see Additional file 3).

### Group comparisons for counseling and treatment service preferences

To reveal differences between symptom groups, a one-way ANCOVA was calculated. The symptom groups did not differ significantly in the mean of counseling and treatment service preferences, when no bootstrapping was applied (*F*(3, 1991) = 2.164, *p* =.090, partial η² = 0.003). However, when bootstrapping was applied, Bonferroni-corrected post-hoc analyses revealed a significant difference between women with PPD symptoms and women without PPD and PAD symptoms (*p* =.040, *M*_Diff_ = −1.144, 95%-CI[−2.271, − 0.027]), but not for the other groups (see Table 4). As shown in Table 4, with an average of 56.3 (*SD* = 8.1), the total score of women with PPD symptoms was lower than that of women without symptoms indicating that they preferred all services less (*M* = 57.4, *SD* = 7.6). More detailed information is provided in an additional file (see Additional file 4).

**Table 4:** Descriptive statistics of the (sub-)scores of questionnaires and differences of symptom groups in the (sub-)score of support preferences

Outcome variable	Without PPD or PAD	PPD	PAD	Comorbid PPD and PAD	Results of ANCOVA	*p*
	*M* (*SD*)					
EPDS score	4.1 (2.6)	11.9 (2.3)	6.8 (2.3)	14.9 (3.7)		
SCL-90-R anxiety score	1.6 (1.7)	3.7 (2.2)	11.0 (3.3)	12.8 (4.5)		
Service preferences ^a^	57.4 (7.6)	56.3 (8.1)	58.5 (7.0)	56.4 (7.9)	PPD < Without ^c^	.040*
Mode preferences ^b^	14.0 (3.4)	14.4 (3.6)	13.6 (3.2)	14.0 (3.7)		n.s.
Counseling and treatment service preferences						
Professional and personal confidants	3.5 (0.4)	3.4 (0.5)	3.6 (0.4)	3.3 (0.5)	Comorbid ^d^< Without ^c^	.002*
					Comorbid ^d^< PPD	.043*
					Comorbid^d^ < PAD	.008*
Communal and psychosocial services	2.7 (0.5)	2.6 (0.6)	2.7 (0.5)	2.6 (0.5)		n.s.
Medical services	3.1 (0.7)	3.0 (0.7)	2.9 (0.8)	3.1 (0.7)		n.s.
Psychotherapeutic services	2.8 (0.7)	2.7 (0.7)	3.0 (0.7)	2.9 (0.7)	PPD < Without ^c^	.022*
					PPD < PAD	.020*
					PPD < Comorbid^d^	.037*
Service delivery mode preferences						
Direct communication	2.6 (0.7)	2.5 (0.7)	2.5 (0.7)	2.6 (0.8)		n.s.
Delayed communication	1.7 (0.5)	1.8 (0.6)	1.7 (0.5)	1.7 (0.6)		n.s.

Note. Bootstrap results are based on 5,000 bootstrap samples

^a^ Counseling and treatment service preferences

^b^ Service delivery mode preferences

^c^ PPD or PAD

^d^ PPD and PAD

* p <.05, two-tailed

### Group comparisons for subscales of counseling and treatment service preferences

A one-way MANCOVA was applied to explore differences between the symptom groups on the subscales of counseling and treatment service preferences (*F*(12, 5220) = 2.618, *p* =.002, partial η² = 0.005, Wilk’s Λ = 0.984). A statistically significant difference between symptom groups could be found for the subscales professional and personal confidants (*F*(3, 1976) = 5.109, *p* =.002, partial η² = 0.008) and psychotherapeutic services (*F*(3, 1976) = 2.996, *p* =.030, partial η² = 0.005). For that reason, for each, a one-way ANCOVA was performed. In both subscales, professional and personal confidants (*F*(3, 2015) = 5.153, *p* =.002, partial η² = 0.008) and psychotherapeutic services (*F*(3, 2009) = 3.261, *p* =.021, partial η² = 0.005), the symptom groups differed significantly.

Bonferroni-corrected post-hoc analyses with bootstrapping were performed to detect differences between symptom groups (see Table 4). Women with comorbid PPD and PAD symptoms rated professional and personal confidants less favorable than women without PPD or PAD symptoms (*p* =.002, *M*_Diff_ = − 0.169, 95%-CI[−0.270, − 0.073]), women with PPD symptoms (*p* =.043, *M*_Diff_ = − 0.119, 95%-CI[−0.233, − 0.008]), and women with PAD symptoms (*p* =.008, *M*_Diff_ = − 0.258, 95%-CI[−0.438, − 0.070]). Psychotherapeutic services were less preferred by women with PPD than by women without PPD or PAD symptoms (*p* =.022, *M*_Diff_ = − 0.112, 95%-CI[−0.208, − 0.017]), women with PAD symptoms (*p* =.020, *M*_Diff_ = 0.306, 95%CI[0.562, 0.039]), and women with comorbid PPD and PAD symptoms (*p* =.037, *M*_Diff_ = − 0.185, 95%-CI[−0.355, − 0.007]). Differences in the mean values can be seen in Table 4. According to Cohen, the effect sizes were very small for both subscales: Professional and personal confidants (partial η² = 0.008) and psychotherapeutic services (partial η² = 0.005). More detailed information is provided in an additional file (see Additional file 5).

### Group comparisons for (subscales of) service delivery mode preferences

No statistically significant difference in service delivery mode preferences was found for the different symptom groups using one-way ANCOVA (*F*(3, 1997) = 0.841, *p* =.471, partial η² = 0.001). Likewise, using one-way MANCOVA, the symptom groups did not differ significantly in the subscales of service delivery mode preferences (*F*(6, 3992) = 1.254, *p* =.276, partial η² = 0.002, Wilk’s Λ = 0.996). Differences in the mean values of total scores and sub-scores can be seen in Table 4.

### Statistical power of the PAD group

A post hoc power analysis was performed using G*Power 3.1.9.7 to determine the power of the symptom group with the smallest size and the effects that can be detected with it. Two power analyses for F-tests (ANCOVA) indicated that for *n* = 29 participants in the PAD group, operated through a total sample size of *n* = 116 for the four symptom groups, power was 82% with Cohen’s ƒ = 0.32. The same results were obtained for one covariate (counseling and treatment service preferences) and two covariates (service delivery mode preferences). Thus, within the PAD group, medium (ƒ = 0.25) to large (ƒ = 0.40) effects can be detected with 80% (1-beta) power and alpha set at 0.05.

In addition, power analyses were conducted for F-tests (MANOVA) since a power analysis for MANCOVA was not readily available via G*Power. The power for the PAD group with *n* = 29 participants was 81% for Cohen’s ƒ^2^ = 0.11 (subscales of counseling and treatment service preferences) and 81% for Cohen’s ƒ^2^ = 0.08 (subscales of service delivery mode preferences). Within the PAD group, effects ranging from small (ƒ^2^ ≥ 0.02) to medium (ƒ^2^ ≥ 0.15) can be detected with 80% (1-beta) power and alpha set at 0.05. Since the statistical power for a MANCOVA should be lower and the required sample size should be larger than for a MANOVA, we assume medium effects for the PAD group within the MANCOVA analyses.

## Discussion

The aim of this study was to contribute to a better understanding of women’s help-seeking behavior in the postpartum period by exploring preferences for various counseling and treatment services and service delivery modes. Key findings were as follows:

 (1) Professional and personal confidants (e.g., *midwife* and *family member*,* friend*,* or colleague*) and direct communication (i.e., *video conference* and *telephone call*) were the most popular services (2), overall, women with PPD preferred all services less than women experiencing neither PPD nor PAD (3), women with comorbid PPD and PAD preferred professional and personal confidants, and (4) women with PPD preferred psychotherapeutic services (e.g., *day clinic* or *outpatient clinic/treatment*) less than all other women.

### Most and least preferred services and modes

In line with previous findings, all mothers, regardless of their symptomatology, preferred to confide in their social environment (e.g., family and friends) or other women suffering from PPD or PAD when experiencing emotional difficulties after giving birth [18, 37, 39]. Hearing other women’s experiences may help correct unrealistic expectations of motherhood and overcome the feeling of not being normal and alone in this [35]. However, the most popular individual services were *midwives and family midwives*, which have rarely been explored in prior studies. In the UK, specialist maternal mental health midwives offer specialized support for mental health problems in addition to standard midwifery care, and work to coordinate and facilitate care pathways [59]. In Germany, postpartum care provided by (family) midwives is an outreach service and is thus exceptional in the German healthcare system. (Family) midwives in Germany help women grow into their role as a mother. Furthermore, the same (family) midwife often provides the entire pre- and postnatal care and thus becomes a confidant for the mother. Prenatal care may also be provided by gynecologists or both. This special role of accompanying women in the perinatal period is also reflected in the descriptive findings where *midwife* and *family midwife* were the most favoured services among all women. Consequently, *(family) midwives* were assigned to professional and personal confidants in this study. In addition to social contacts, peers, and (family) midwives, *gynecologists* were nearly as popular. In Germany, many women know their gynecologist from regular visits during pregnancy and have often developed a trusting relationship. It is likely that trust and security play an important role when women discuss sensitive issues such as depressive or anxiety symptoms and that they are more likely to experience this with familiar rather than unknown providers [26]. This is usually the case with (family) midwives and gynecologists in Germany.

One service that women were reluctant to use was *inpatient clinic for psychiatry or psychosomatic medicine*. Compared to inpatient treatment, however, outpatient treatment was rated very positively by mothers. Possible reasons could be the medication often associated with inpatient stays, potential time apart from the infant (since some clinics do not allow the child to be present), and insufficient childcare for older children. Studies have shown that medication for PPD and PAD is, especially in terms of breastfeeding, unpopular among mothers [21, 33, 60, 61]. Overall, the groups differed in their preferred type of therapy. On the whole, women preferred mother-child therapy over therapy of the mother only. However, this trend was mainly seen for women with no or subliminal psychopathological symptoms. Especially women with comorbid PPD and PAD symptoms liked both options almost equally. Nevertheless, the overall preference for mother-child therapy may be related to the argument of separation from the infant.

In terms of service delivery mode, *in person* was by far the most popular. This finding is consistent with previous research [33, 36]. Overall, there was a clear tendency towards direct communication (i.e., *video conference* and *telephone call*) rather than delayed communication (comprising web-based interventions). Previous studies have also shown that e-mental health interventions tend to be unpopular [33, 38–40].

### Group differences for counseling and treatment service preferences

Overall, women with PPD preferred all services less than women with neither PPD nor PAD. A possible explanation is that the symptomatology of PPD acts as a barrier. Women with PPD may experience symptoms such as negative perceptions of the self and their environment, self-stigmatization (e.g., being a “bad mother”), avolition, or hopelessness [62–65]. This may lead to less willingness to (actively) seek help, more negative attitudes toward services, and doubts about whether treatment is helpful [66].

Women with PPD preferred psychotherapeutic services (e.g., *day clinic* or *outpatient clinic/treatment*) less than all other women. There is a lot of stigma associated with depression, especially for women in the postpartum period [67], and it is still often equated with being a “bad mother”. This mindset may still prevail in Germany as well. Experiencing symptoms of anxiety in the postpartum period, especially if you are a first-time mother, may be considered more normal and less stigmatized. For example, it seems more understandable to be overly concerned about the infant than to be depressed as a mother. Several studies have shown that fear of stigma is one of the most important barriers to help-seeking among women with PPD [20, 34, 68]. In particular, the use of psychotherapeutic services would reveal that the women are suffering from depressive symptoms that they may wish to conceal from those around them. Furthermore, unlike the UK, for example, there are no nationwide perinatal psychotherapeutic services available, especially outside the major cities, and there are long waiting lists, and a lack of inpatient services with child care in Germany. This is especially challenging for depressed women with little drive and energy. However, this cannot explain the difference between women with PPD and women with comorbid PPD and PAD, and further research is needed.

Women experiencing comorbid PPD and PAD symptoms preferred support from professional (i.e., *(family) midwife*) and personal (i.e., *family*,* friend*,* or colleague* or *woman in the same situation*) confidants less than all other women. Several reasons for this effect can be inferred. As previously mentioned, symptom severity is greater in women with comorbid symptoms of depression and anxiety [8, 9]. One possibility is that women may be particularly afraid of disclosing their symptoms to those around them and possibly being misunderstood or even rejected [36, 68]. However, it is also conceivable that women did not want their suffering to be a burden to their environment [69]. Finally, women may recognize the severity of their symptoms and the necessity of professional help (e.g., counselor or psychotherapist). One study showed that women with PPD were less likely to seek informal help, but more likely to seek professional help when they recognized their need for treatment [18]. This may be even more true for women with both PPD and PAD.

### Group differences for service delivery mode preferences

All women rated the service delivery mode equally overall. They showed the same pattern in their preferences for direct and delayed communication. These findings suggest that the delivery mode does not appear to be symptom-specific, and the advantages and disadvantages of the different delivery modes apply to all women.

### Implications for practice

Finally, several practical implications can be drawn from our results. In this study, it has been shown that women with PPD and women with comorbid PPD and PAD have divergent preferences for different types of services, which should be considered when making referrals. If health professionals know whether and which symptoms are present in postpartum women, they can match treatment to their needs and preferences and refer to a specific support service depending on the type of symptoms. By aligning services with women’s expressed preferences, health care providers can increase engagement and improve outcomes for this vulnerable population. In addition, treatment programs should be tailored to specific symptoms (i.e. depression or anxiety) because they require different decisions (e.g., regarding medication) and forms of treatment. Also, women with subliminal symptoms or women who are at high risk should be involved in healthcare at early stages and receive preventive support. Informing policymakers about preferences can guide attention and funding towards these services. Investing in prevention and providing effective help can lead to reduced long-term healthcare costs [70].

Once detected, women with PPD should be particularly encouraged to seek help and should be thoroughly informed about counseling and treatment options, as overall, they found all services to be the least appealing. In addition, women with PPD were less inclined to psychotherapeutic services than all other women. However, as these women need appropriate treatment, health professionals can try to educate them about the relevance of such services and reduce possible prejudices and barriers. Women with comorbid PPD and PAD should be especially offered professional help, as they are less likely to seek help from professional or personal confidants.

Among all women, *(family) midwives* were the most popular caregiver. As a result, they should be sufficiently trained in perinatal mental health issues to adequately respond and refer women to appropriate providers. Also, *family*,* friends*,* and colleagues* were important support persons to whom women would turn. Therefore, campaigns must target not only the mother herself but also her social environment [39, 71]. Treatment programs could include family members for example by training them on how to provide effective emotional support. It is important that the social environment is educated about the psychopathology and treatment options so that they can further support the mother. Moreover, the strong preference for (family) midwives and support from the social environment underscores the need for health care providers to prioritize these relationships in care plans and facilitate access to the supportive networks that women find most comforting and helpful. Overall, communal and psychosocial services were the least popular type of service, and some individual offers, such as *supervised parent group* and *psychosocial crisis service*, were rather unknown. To determine the potential usefulness of these services and identify any barriers to their popularity, it may be beneficial to advertise them more widely. This could help address issues such as lack of knowledge.

For all women, *in person* was by far the most preferred delivery mode, whereas telephone-based interventions were less popular. Yet, the latter are flexible, non-stigmatizing, and may reduce some barriers to healthcare [72]. Therefore, *telephone call* and *video conference* are important alternative delivery modes that should be increasingly considered, depending on women’s characteristics. Alternative, low-cost, effective, and far-reaching methods of support offered online are important to fill the support gap experienced by many women in the perinatal period [35]. In particular for women with subliminal symptoms, e-mental health can provide help at an early stage. Nonetheless, the potential benefits of e-mental health are not reflected in women’s preferences for service provision in this study. Therefore, e-mental health interventions need to be better adapted to women’s needs. In addition, (mental) healthcare professionals should be more knowledgeable and trained in the use of these novel treatment modes.

### Strengths and limitations

In this study, we examined a large sample of postpartum women. Women were approached in various settings, including high and low risk pregnancies. Therefore, the results can be generalized to women in different birth settings. With regard to the method, interviews conducted over the telephone are a valid option to face-to-face interviews, with a high level of agreement found [73, 74]. Compared to face-to-face interviews, positive aspects are the decreased burden (e.g., staying at home) and increased anonymity [23], which may make it easier for new mothers to share sensitive information. Another contribution of this study is the wide range of services offered by different providers that were surveyed. In addition, there are few to no studies that have compared the individual symptom groups of PPD, PAD, comorbid PPD and PAD, and women with neither PPD nor PAD.

Despite its important contribution, this study has some limitations that must be considered. Our sample mainly comprised women with above-average levels of education and income compared to the general German [75] and Dresden population [76]. Selection bias could account for the demographic skew, as people with higher socioeconomic status may be more likely to participate in research [77, 78]. In addition, the vast majority of women were born in Germany. Compared to the population of Dresden, the proportion of people with German citizenship or without a migration background is only slightly higher (88%, [79]). Therefore, caution should be exercised regarding the generalizability of the study results as conclusions drawn from the study may not be applicable to broader populations, particularly those facing different socioeconomic challenges and with more diverse cultural backgrounds. Therefore, caution should be taken in generalizing to other populations. Prevalence estimates for PAD should be viewed with caution due to the small size of the PAD symptom group in this study, which only had statistical power to detect medium to large effects. In contrast, the field of psychology often yields effect sizes that are relatively small [80]. It should also be noted that the study took place during different phases of the Covid-19 pandemic and it cannot be ruled out that exceptional situations (such as the lockdowns) may have had an impact. First, it is expected that the response rate was lower during the lockdown periods because the student assistants were not able to contact the women personally and therefore the planned recruitment strategy could not be implemented. In addition, the pandemic may have exacerbated the mental health problems of many postpartum women, who may have had less mental capacity to participate in research. Second, the pandemic may also have influenced women’s preferences. For example, many services, especially in the communal and psychosocial sectors, were either not accessible or offered at a reduced capacity to women during some parts of the study. Many women may have felt frustrated or unsupported by the temporary closure, restrictions, and lack of accessible options, and thus preferred other forms of support. Reliabilities for the subscales medical services and professional and personal confidants were questionable and unacceptable, respectively. For the subscale direct communication, McDonald’s ω could not be calculated. In subsequent research, they should be reviewed for quality.

### Implications for future research

Although in line with previous large epidemiological studies [78, 81, 82], future research should investigate if the trends in this study can be replicated to women with lower socio economic status. Moreover, replications should be performed with a symptom group that contains more anxious mothers. It remains interesting to examine the extent to which previous counseling and treatment experiences have an impact on women’s current preferences. Past experiences were found to be a consistent predictor of help-seeking attitudes [42, 83], and therefore it should also be investigated how awareness of services affects preferences.

## Conclusions

This study addressed knowledge gaps related to counseling and treatment service and service delivery mode preferences among postpartum women. Our results showed that women differ in their preferences for services depending on their symptoms, but not in their preferences for delivery mode. Therefore, support services should be tailored to women’s needs and preferences, and specific referrals should be made to improve their help-seeking behavior. This will ultimately help decrease long-term healthcare expenses, for example by reducing the use of health care services, which are very expensive for health insurers, or by reducing unemployment due to mental disorders [70]. Overall, there is a need to increase awareness of the symptoms of PPD and PAD and knowledge of the different support options. In addition, efforts should be made to make the field of e-mental health a more popular option for postpartum women, as it is time and cost effective and can be helpful while waiting for or accompanying in-person treatment. Even though the German healthcare system and services differ from those in other countries, general recommendations can be derived for the support of postpartum women to improve their mental health.

## Supplementary Information

Below is the link to the electronic supplementary material.

Supplementary Material 1.

Supplementary Material 2.

Supplementary Material 3.

Supplementary Material 4.

Supplementary Material 5

## Data Availability

The datasets generated and analysed during the current study are not publicly available due to legal and ethical constraints. Public sharing of participant data was not included in the informed consent of the study. Requests to access the datasets should be directed to Susan Garthus-Niegel, susan.garthus-niegel@uniklinikum-dresden.de.

## Abbreviations

ANCOVA: Analysis of covariance
EPDS: Edinburgh Postpartum Depression Scale
IQR: Interquartile range
INVITE: INtimate partner VIolence Treatment PrEferences
MANCOVA: Multivariate analysis of covariance
MDD: Major depressive disorder
PAD: Postpartum anxiety disorder
PFA: Principal Axes Factor Analysis
PPD: Postpartum depression
REDCap: Research Electronic Data Capture
SCL-90-R: Symptom-Checklist-90-Revised
