# Supplementary material for: Support preferences among women with and without postpartum depression and anxiety disorder
Source: BMC Public Health. 2025 Sep 12;25:3048. doi: 10.1186/s12889-025-24274-y (PMC12427099; doi:10.1186/s12889-025-24274-y)
Supplement: Supplementary file 1 — Supplementary Material 1. [file 12889_2025_24274_MOESM1_ESM.pdf]

Additional file 1

Rotated factor matrix of principal axes factor analysis (PFA) for the items of counseling and treatment service preferences

Item	Factor loading			
	1	2	3	4
**Factor 1: Professional and personal confidants**				
03. Woman in the same situation	**.480**	.134	.042	-.006
02. Family member, friend, or colleague	**.442**	.000	.014	.096
14. Midwife	**.369**	.071	.369	.068
09. Family midwife	**.300**	.334	.060	.088
**Factor 2: Communal and psychosocial services**				
11. Social pedagogical family assistance	-.006	**.685**	.082	.214
08. Parent-child living or family accommodation	-.025	**.622**	.089	.133
10. Psychosocial crisis service	.061	**.600**	-.003	.311
07. Life and family counseling center	.044	**.577**	.064	.132
05. Supervised parent group	.197	**.493**	.027	.103
06. Telephone counseling	-.014	**.479**	.050	.047
01. Self-help group	.159	**.402**	.053	.080
04. Household help	.125	**.368**	.023	.135
**Factor 3: Medical services**				
13. Pediatrician	-.042	.088	**.726**	.012
15. Gynecologist	.122	.046	**.588**	.063
12. General practitioner	.025	.063	**.564**	.152
**Factor 4: Psychotherapeutic services**				
17. Day clinic for psychiatry or psychosomatic medicine	-.008	.262	.130	**.783**
18. Outpatient clinic/treatment for psychiatry, psychosomatic medicine, or psychotherapy	.178	.153	.044	**.699**
16. Inpatient clinic for psychiatry or psychosomatic medicine	-.036	.244	.175	**.680**
19. Specialized trauma outpatient clinic	.246	.287	.056	**.560**

Note. Extraction method: Principal Axes Factor Analysis using Varimax Rotation with fixed number of factors (4) and Kaiser Normalization, one item (religious institutions) was excluded from analysis.
