# Supplementary material for: Support preferences among women with and without postpartum depression and anxiety disorder
Source: BMC Public Health. 2025 Sep 12;25:3048. doi: 10.1186/s12889-025-24274-y (PMC12427099; doi:10.1186/s12889-025-24274-y)
Supplement: Supplementary file 2 — Supplementary Material 2. [file 12889_2025_24274_MOESM2_ESM.pdf]

**Additional file 2**

Rotated factor matrix of principal axes factor analysis (PFA) for the items of service delivery mode preferences

| Item | Factor loading | |
|---|---|---|
| | 1 | 2 |
| **Factor 1: Direct communication** | | |
| 03. Telephone call | **.801** | .224 |
| 02. Video conference | **.516** | .086 |
| **Factor 2: Delayed communication** | | |
| 06. App or online platform without guidance from expert | .057 | **.739** |
| 05. App or online platform with guidance from expert | .141 | **.711** |
| 04. E-mail | .255 | **.631** |
| 03. Chat | .197 | **.618** |

Note. Extraction method: Principal Axes Factor Analysis using Varimax Rotation with Kaiser Normalization, one item (in person) was excluded from analysis.
