# Supplementary material for: Support preferences among women with and without postpartum depression and anxiety disorder
Source: BMC Public Health. 2025 Sep 12;25:3048. doi: 10.1186/s12889-025-24274-y (PMC12427099; doi:10.1186/s12889-025-24274-y)
Supplement: Supplementary file 3 — Supplementary Material 3. [file 12889_2025_24274_MOESM3_ESM.pdf]

Additional file 3

Spearman correlation matrix including all potential confounders and outcome variables

Variable	1	2	3	4	5	6	7	8	9	10	11	12
1. Maternal age	–											
2. Residence in GER [a]	.018	–										
3. Income	.212*	.040	–									
4. Parity	.320*	.034	.043	–								
5. Service preferences [b]	**.048***	0,018	.032	.008	–							
6. Mode preferences [c]	**.059***	-0,029	.031	**-.062***	.134*	–						
7. Confidants [d]	.019	,132*	.098*	-.098*	.489*	.082*	–					
8. Communal and psychosocial [e]	.041	-.044	-.013	.034	.826*	.145*	.242*	–				
9.Medical [e]	.041	.001	-.005	.014	.471*	.025	.192*	.151*	–			
10. Psychotherapeutic [e]	.047*	.026	.048*	-.004	.695*	.053*	.211*	.428*	.211*	–		
11. Direct [f]	.066*	.011	.119*	-.066*	.165*	.662*	.103*	.153*	.021*	.129*	–	
12. Delayed [f]	.044*	-.041	-.024	-.042	.088*	.896*	.041	.108*	.031	.001	.287*	–

Note. Significant correlations between confounders (maternal age, duration of residence in Germany, income, parity) and outcome variables (counseling and treatment service preferences and service delivery mode preferences) are printed in bold.

[a] Duration of residence in Germany, [b] Counseling and treatment service preferences, [c] Service delivery mode preferences, [d] Professional and personal confidants, [e] Services, [f] Communication.

* $p < .05$.
