# Supplementary material for: Support preferences among women with and without postpartum depression and anxiety disorder
Source: BMC Public Health. 2025 Sep 12;25:3048. doi: 10.1186/s12889-025-24274-y (PMC12427099; doi:10.1186/s12889-025-24274-y)
Supplement: Supplementary file 4 — Supplementary Material 4. [file 12889_2025_24274_MOESM4_ESM.pdf]

Additional file 4

Differences of symptom groups in the total score of counseling and treatment service preferences

(I) Comparing symptom groups	(J) Comparing symptom groups	Mean difference (I–J)	*p*	95% CI	
				LL	UL
Without PPD or PAD	PPD	1.144	**.040***	.073	2.224
	PAD	-1.060	.399	-3.694	1.547
	Comorbid [a]	1.094	.215	-.666	2.792
PPD	Without [b]	-1.144	**.040***	-2.271	-.027
	PAD	-2.204	.106	-5.008	.559
	Comorbid [a]	-.050	.962	-2.089	1.981
PAD	Without [b]	1.060	.399	-1.422	3.519
	PPD	2.204	.106	-.431	4.857
	Comorbid [a]	2.154	.154	-.926	5.107
Comorbid PPD and PAD	Without [b]	-1.094	.215	-2.761	.610
	PPD	.050	.962	-1.895	1.994
	PAD	-2.154	.154	-5.159	.997

Note. Bootstrap results are based on 5,000 bootstrap samples. CI = bias-corrected and accelerated bootstrap interval, LL = lower limit, UL = upper limit.

[a] PPD and PAD, [b] PPD or PAD.

* $p < .05$, two-tailed.
