# Supplementary material for: Support preferences among women with and without postpartum depression and anxiety disorder
Source: BMC Public Health. 2025 Sep 12;25:3048. doi: 10.1186/s12889-025-24274-y (PMC12427099; doi:10.1186/s12889-025-24274-y)
Supplement: Supplementary file 5 — Supplementary Material 5 [file 12889_2025_24274_MOESM5_ESM.pdf]

Additional file 5

Differences of symptom groups in the sub-scores of counseling and treatment service preferences

(I) Comparing symptom groups	(J) Comparing symptom groups	Mean difference (I–J)	*p*	95% CI	
				LL	UL
**Professional and personal confidants**					
Without PPD or PAD	PPD	.051	.134	-.012	.118
	PAD	-.089	.283	-.241	.083
	Comorbid [a]	.169	**.002***	.071	.272
PPD	Without [b]	-.051	.134	-.120	.014
	PAD	-.140	.111	-.306	.035
	Comorbid [a]	.119	**.043***	.002	.240
PAD	Without b	.089	.283	-.092	.247
	PPD	.140	.111	-.045	.313
	Comorbid [a]	.258	**.008***	.063	.445
Comorbid PPD and PAD	Without [b]	-.169	**.002***	-.270	-.073
	PPD	-.119	**.043***	-.233	-.008
	PAD	-.258	**.008***	-.438	-.070
**Psychotherapeutic services**					
Without PPD or PAD	PPD	.112	**.022***	.015	.208
	PAD	-.193	.126	-.439	.059
	Comorbid [a]	-.073	.343	-.220	.080
PPD	Without [b]	-.112	**.022***	-.208	-.017
	PAD	-.306	.020*	-.562	-.039
	Comorbid [a]	-.185	**.037***	-.355	.007
PAD	Without [b]	.193	.126	-.059	.438
	PPD	.306	**.020***	.038	.563
	Comorbid [a]	.121	.416	-.170	.403
Comorbid PPD and PAD	Without [b]	.073	.343	-.080	.220
	PPD	.185	**.037***	.006	.357
	PAD	-.121	.416	-.417	.179

Note. Bootstrap results are based on 5,000 bootstrap samples. CI = bias-corrected and accelerated bootstrap interval, LL = lower limit, UL = upper limit.

[a] PPD and PAD, [b] PPD or PAD.

* $p < .05$, two-tailed.
